# Exploring cell-free assays for COVID-19 serosurvey

**DOI:** 10.1038/s41598-024-55852-6

**Published:** 2024-03-13

**Authors:** Lucia Inchauste, Elif Nurtop, Nadège Brisbarre, Laetitia Ninove, Pierre Gallian, Xavier de Lamballerie, Stéphane Priet

**Affiliations:** 1 Unité des Virus Émergents (UVE: Aix-Marseille Univ, Università di Corsica, IRD 190, Inserm 1207, IRBA), Marseille, France; 2 Établissement Français du Sang Provence Alpes Côte d’Azur et Corse, Marseille, France; 3 Établissement Français du Sang, La Plaine Saint-Denis, Saint-Denis, France

**Keywords:** SARS-CoV-2, Infectious-disease diagnostics, Viral infection

## Abstract

Serosurveys to monitor immunity toward COVID-19 in the population are primarily performed using an ELISA to screen samples for SARS-CoV-2 antibodies, followed by confirmation by a virus neutralization test, which is considered the Gold Standard. However, virus neutralization test may not be feasible for some laboratories because of the requirement for specific facilities and trained personnel. In an attempt to address this limitation, we evaluated three cell-free methods as potential alternatives for assessing SARS-CoV-2 seroprevalence in human population from plasma. We report the establishment of two inhibition ELISAs designed to detect anti-Spike RBD IgG antibodies and a microsphere quantitative suspension array technology assay, based on the Luminex xMAP platform, to measure the presence of antibodies against various SARS-CoV-2 antigens, including anti-RBD. These methods were also compared to a commercial chemiluminescent immunoassay designed for anti-RBD antibodies detection and to the combined ELISA + virus neutralization test strategy. These cell-free assays performed equally to estimate the percentage of positive and negative samples and could be used to determine the prevalence of SARS-CoV-2 antibodies in human population, at least in cohort with high-expected prevalence, without the use of seroneutralization assay.

## Introduction

COVID-19 is a viral infection caused by SARS Coronavirus 2 (SARS-CoV-2), first identified in December 2019 in Wuhan, Hubei Province, China^1,2^. This virus belongs to the* Coronaviridae* family, *Orthocoronavirinae* subfamily, genus *Betacoronavirus* among a total of four genera: *Alphacoronavirus* and *Betacoronavirus*, that infect various mammalian species, and *Gammacoronavirus* and *Deltacoronavirus*, that infect birds^3,4^. To date, seven coronaviruses are known to infect humans, including the human coronaviruses (HCoVs) known as common cold viruses, HCoV-OC43, HCoV-229E, HCoV-NL63, and HCoV-HKU1 described in 1966, 1967, 2004, and 2005, respectively^5–8^. SARS-CoV and MERS-CoV were discovered in 2003 and 2012, respectively, and are responsible for high mortality^9,10^. The SARS-CoV outbreak affected at least 8000 people with a case fatality rate of about 15%, while MERS-CoV caused at least 2000 cases with a case fatality rate of about 35%^11,12^. Regarding the SARS-CoV-2 pandemic, as of January 3, 2023, there have been 661,439,590 cases in 192 countries and more than 6,693,057 deaths from at least 8 waves following the emergence of variants^13^.

All coronaviruses share four structural proteins: the spike glycoprotein (S), the membrane glycoprotein M, the envelope protein (E), and the nucleoprotein (N)^3^. The Spike is a transmembrane homotrimer composed of two subunits: S1, which for both SARS-CoV and SARS-CoV-2 mediates viral attachment to the cellular angiotensin-converting enzyme 2 (ACE2) receptor through its receptor-binding domain (RBD), and S2, which mediates membrane fusion^14,15^. The S1 subunit is composed of four domains: the N-terminal domain (S-NTD), the receptor-binding domain (RBD), and two structurally conserved subdomains (SD1 and SD2) at the C-terminus. The S1 subunit exhibits two different conformations of its RBD domain, allowing for adjustment of accessibility to ACE2^16,17^. In the pre-fusion state of the Spike homotrimer, two RBDs are in a ‘down’ state and one is in an ‘up’ state. The latter represents a receptor-accessible state that allows binding to ACE2^15,18^. As Spike mediates host cell attachment and entry, it is naturally the main target of neutralizing antibodies (nAbs)^19^. Within the Spike, RBD is the major immunodominant site, being the target of 90% of nAbs, followed by S-NTD and others quaternary epitopes on the Spike trimer or the S2 subunit^20–22^. Five major epitopes within the RBD have been reported to date, and three of them fully or partially overlap with the ACE2 binding site, also called the Receptor Binding Motif (RBM)^23–25^. nAbs can only access the RBD in the ‘up’ state for epitopes that fully overlap the RBM, whereas those directed to partially overlapping epitopes can access the RBD in both states^23–25^. A fourth epitope is located in the left flank of the RBD, also called as the CR3022 cryptic site, and nAbs targeting this zone can only access the RBD in the ‘up’ states^25,26^. Finally, nAbs directed to the right flank of the RBD can access it in both the ‘up’ and ‘down’ states^25,26^. In terms of cross-reactivity, since the SARS-CoV-2 Spike protein shares 77.5% sequence identity with the SARS-CoV Spike, some cross-reaction is observed for nAbs^23,25^. The percentage of cross-reaction drops to 1–6% for the other coronaviruses, as they are more distantly related (sequence identity ranging from 25 to 30% for common cold coronaviruses and 31% for MERS CoV)^23^. However, for antibodies that recognize the RBD domain, cross-reactivity is observed only with SARS-CoV, as it shares 74% sequence identity, and not with other coronaviruses, whose sequence identity ranges only from 13 to 21%^23,25^.

Seroprevalence studies are complementary to active surveillance and allow analysis of the level of immunity of a population to a given pathogen without the need for testing during the short period when patients are symptomatic^27^. Moreover, cross-sectional seroprevalence studies can help to more accurately determine the rate of infection, case-fatality rate, and assess herd immunity and humoral protective immunity^28,29^. Various methods exist for assessing the presence of antibodies (IgG, IgM, or IgA) produced as a result of host immune responses to pathogen infection or vaccination, including virus neutralization tests (VNT), immunoassays such as enzyme-linked immunosorbent assays (ELISA) or chemiluminescent immunoassay (CLIA), and rapid lateral flow tests. Cell-free assays such as ELISA and related techniques allow testing rapidly a large number of samples in parallel in a standard laboratory. Most conventional ELISAs detect reactive immunoglobulins but do not assess the presence or absence of neutralizing antibodies that are considered less likely to cross-react with related pathogens (e.g., other coronaviruses in case of SARS-CoV-2) and are known to overestimate the real infection status^30^. In contrast, as it is supposed to have the best specificity, the gold standard method to date is VNT. VNT has also the great advantage of detecting functional antibodies (i.e. neutralizing antibodies) against a specific pathogen, which allow inferring protective immunity after vaccination or infection. However, this method requires biosafety laboratories of level 2 or 3 to perform the culture of pseudo-typed or wild-type viruses^31^, is difficult to implement at high throughput and is time consuming. Therefore, a convenient strategy (referred to below as “ELISA + VNT” strategy) is to screen samples by anti-Spike S1 ELISA, followed by VNT confirmation of the ELISA non-negatives samples^30–32^.

Here, we evaluated three cell-free methods as potential alternatives to assess the seroprevalence of SARS-CoV-2 in human population from plasma. We first modified two commercial anti-Spike S1 ELISAs to create inhibition/competition assays allowing the detection of anti-RBD IgG antibodies. We also established and validated a test using the microsphere quantitative suspension array technology (qSAT) on the Luminex xMAP platform to measure the presence of IgG antibodies against various SARS-CoV-2 antigens. The commercial Access SARS-CoV-2 IgG II test that detects anti-RBD IgG antibodies from Beckman Coulter was also included in the analyzes. The performances, limitations, and advantages of these assays are reported in comparison with anti-Spike S1 ELISA screening followed by VNT confirmation.

## Results

### Study samples

To evaluate the performances of the serological assays described hereafter, we used an Epidemic panel (cf. Methods) containing 486 samples collected in France before the vaccination campaign. This panel was characterized by the “ELISA + VNT” strategy where the status of each sample was defined as positive when the VNT result was positive and/or both anti-S1 and -NCP ELISAs were positive. The panel was composed of 280 (57.6%) positive and 206 (42.4%) negative samples (Supplementary Table 1). Each sample was tested for anti-SARS-CoV-2 Spike RBD IgG by inhibition/competition ELISAs (see below iELISA and iqELISA) and Beckman Access SARS-CoV-2 IgG II Immunoassay and for IgG directed against Spike, RBD domain and Nucleoprotein using a quantitative suspension array technology (qSAT) microsphere assay.

### Validation and assay performance indicators for a panel of serological tests

#### iELISA and iqELISA

The Epidemic panel was tested with both iELISA and iqELISA, and the percentage of inhibition (%Inhibition) was calculated relative to the results (ratio or BAU/mL for iELISA and iqELISA, respectively) obtained without the incubation of recombinant RBD protein prior to ELISA (see details in methods).

**Figure 1:**
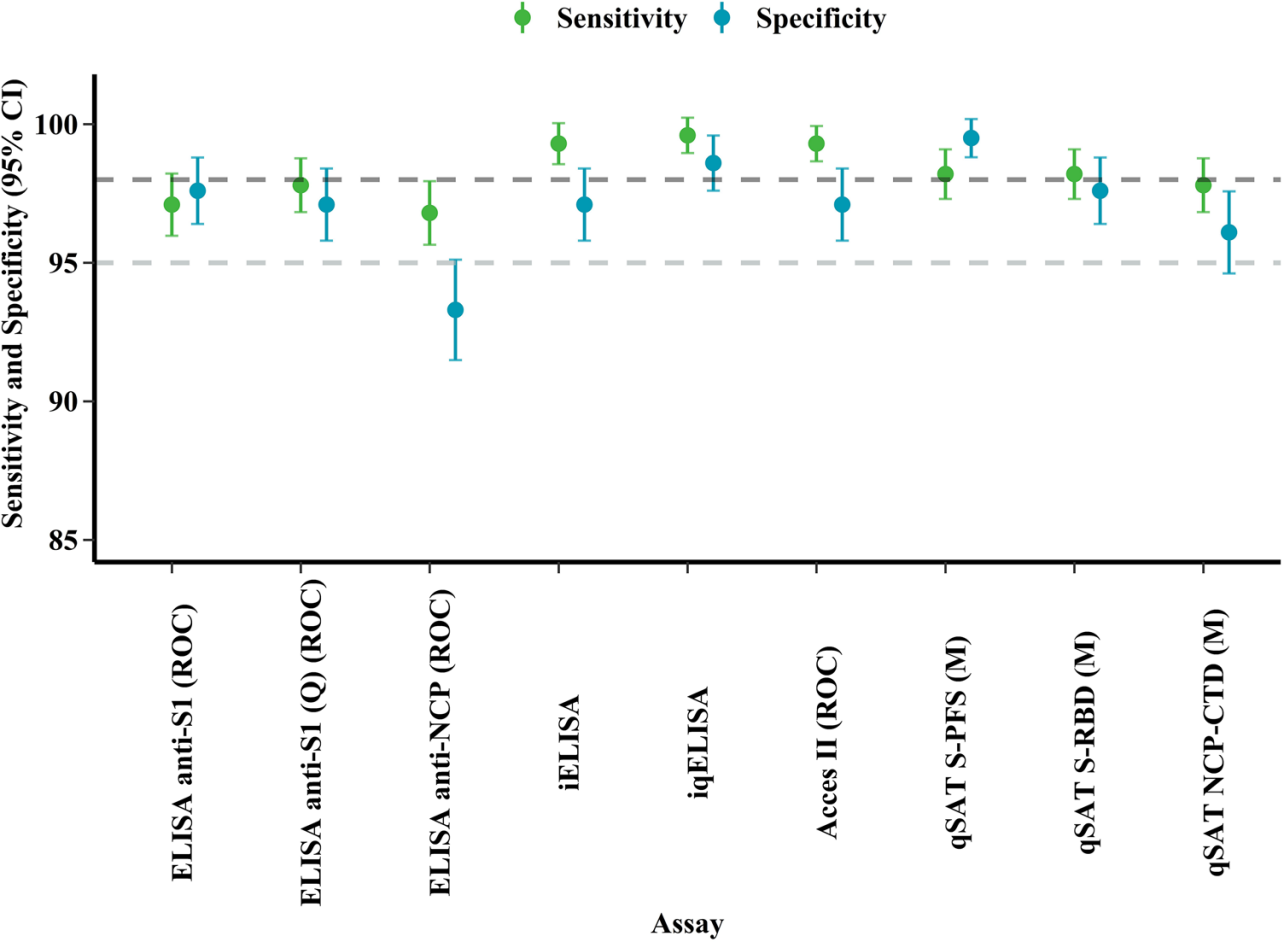
Empirical test performance indicators. Sensitivity (green dots), specificity (lightblue dots) and respective 95% confidence intervals (95% CI, error bars) were calculated by receiver operating characteristic curve (ROC) analysis.

The empirical cut-off value and test performance indicators (sensitivity and specificity) for iELISA and iqELISA were first calculated by Receiver Operating Characteristic curve (ROC) analysis using the %Inhibition, corr %Inhibition, and the status (positive and negative) for each sample (Fig. 1, Supplementary Fig. S1 and Supplementary Table 1). The percentage of positive (58.2% vs. 57.8%) and negative (41.8 vs. 42.2%) samples, as well as the sensitivity (Se) and specificity (Sp) of the iELISA (Se: 99.3%, 95% CI 96.9–99.8; Sp: 97.1%, 95% CI 93.8–98.9) and iqELISA (Se: 99.6%, 95% CI 97.4–99.9; Sp: 98.6%, 95% CI 95.8–99.7) were similar.

To further assess the specificity of both iELISA and iqELISA, we analyzed the Pre-epidemic panel composed of 180 plasma samples (cf. Methods). Using the threshold established by the ROC curve analysis, the specificities were 98.9%, and 98.3% for the iELISA and iqELISA, respectively.

#### Beckman access SARS-CoV-2 IgG II assay

Anti-RDB antibodies were also assessed for each sample in the Epidemic panel with Beckman's commercial Access SARS-CoV-2 IgG II assay. The cut-off value of 30 IU/mL (Acces II (Beckman)) recommended by the manufacturer led to a sensitivity of 92.5% (95% CI 88.7–95.3) and a specificity of 99.0% (95% CI 96.6–99.9). We also calculated the test performances with the cut-off value of 15 IU/mL (Acces II (ROC)) suggested by the ROC analysis, giving a sensitivity of 99.3% (95% CI 97.4–99.9) and a specificity of 97.1% (95% CI 93.8–98.9) (Fig. 1, Supplementary Fig. S1 and Supplementary Table 1).

#### qSAT assay

Considering that Spike and NCP are the most immunogenic proteins in SARS-CoV-2, we have evaluated the performance of these antigens in a Luminex-based qSAT assay. We decided to test both the full length S protein and the RBD, since several epitopes present outside the RBM/RBD were likely to be recognized on the full-spike or only within a trimeric Spike. Preliminary experiments in a singleplex format showed that the full-spike S-PFS in the pre-fusion state displayed the best specificity and that NCP-CTD displayed superior sensitivity and specificity over the NCP-NTD domain (Supplementary Fig. S1 and Supplementary Table 1). All samples of the Epidemic panel were thus analyzed in a multiplex format using the S-PFS, RBD, and NCP-CTD antigens that displayed the best performances in singleplex format. Results correlated with the singleplex format, showing that both were equivalent (Supplementary Fig. S2). Sensitivities were similar for the antigens (98.2%, 95% CI 95.9–99.4 for the S-PFS and the RBD, 97.8%, 95% CI 95.4–99.2 for the NCP-CTD), while the highest specificity was obtained by S-PFS (99.5%, 95% CI 97.3–100), followed by RBD (97.6%, 95% CI 94.5–99.2), and by NCP-CTD (96.1%, 95% CI 92.5–98.3). Results obtained with the S-PFS antigen in MFI or BAU/mL (cf. Methods) are identical (Supplementary Table 1).

To further assess the specificity of qSAT in the multiplex format, we analyzed the Pre-epidemic panel (cf. Methods). Using the threshold established by the ROC curve analysis, the specificities were 99.5%, 99.0% and 99.5% for S-PFS, RBD, and NP-CTD, respectively.

### Correlations among cell-free serological assays

**Figure 2:**
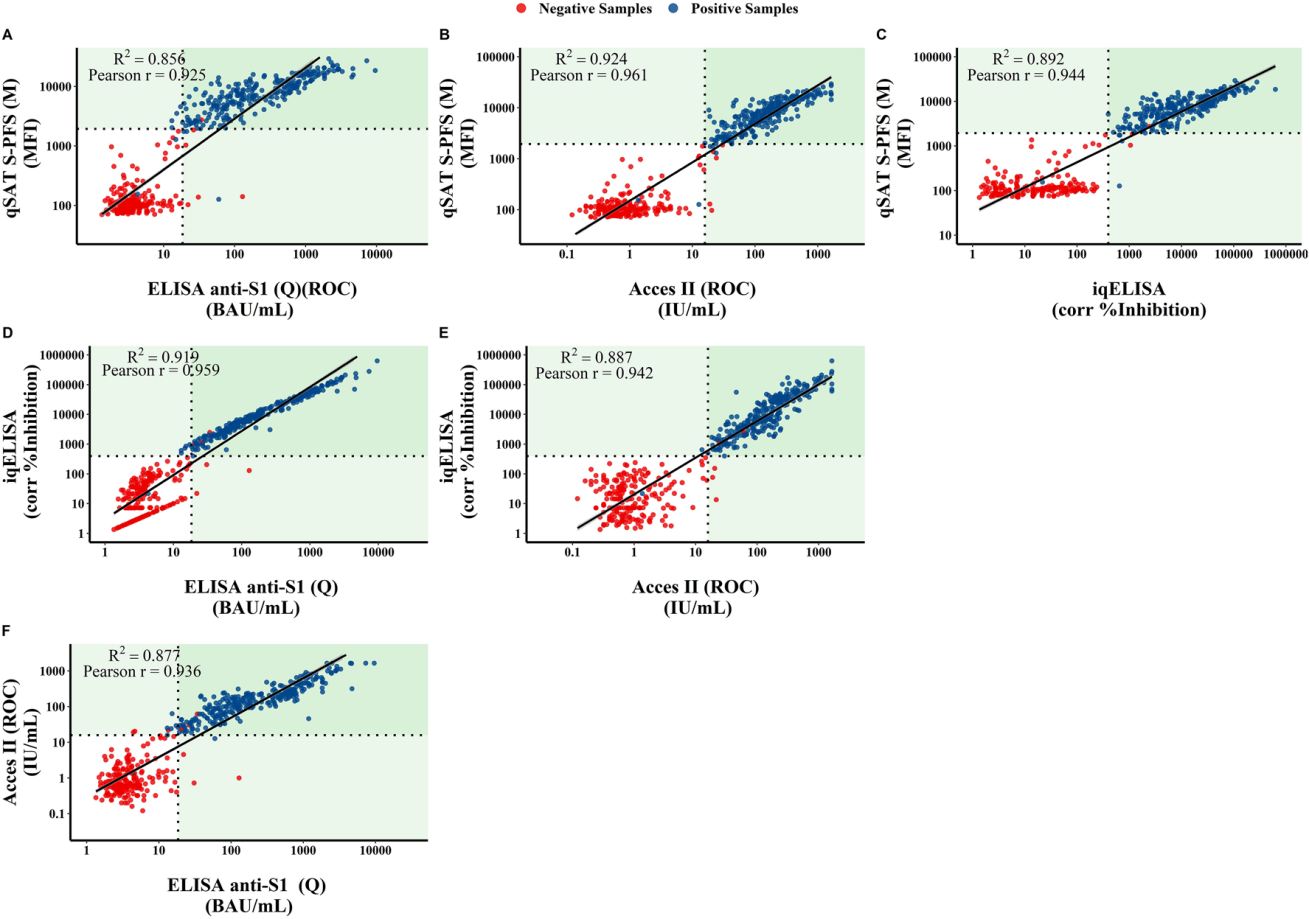
Side-by-side comparisons between all serological assays using the S or RBD antigens. Samples were considered positive (blue dots) with either a positive VNT result or a positive result for both the anti-Spike S1 and -NCP IgG ELISA, others were considered negative (red dots). Side-by-side comparisons were assessed through a Pearson correlation test. *P* value for each comparison was < 0.001.

To gain further insight into test performances, side-by-side comparisons were made for all serological assays using a Pearson correlation test. First, we compared the three qSAT antigens in order to determine the correlation between each other. As expected, higher correlations were observed between S-PFS and RBD (Supplementary Fig. S3). Then comparisons between all the serological tests using the spike-derived antigens were performed (Fig. 2). As all test detected antibodies targeting a region within the Spike, strong positive correlations were found between all tests (Pearson r ranging from 0.852 to 0.924). Comparison of the number of false positive (FP) from false negative (FN) samples, between all tests confirmed that the qSAT assay using the S-PFS antigen and the iELISA/iqELISA proved to be the best serological assays followed by the Beckman Immunoassay and the anti-SARS-CoV-2 IgG S1 ELISA (Fig. 2, Supplementary Table 1).

### Correlation of cell-free serological tests with the SARS-CoV-2 viral neutralization test

**Figure 3:**
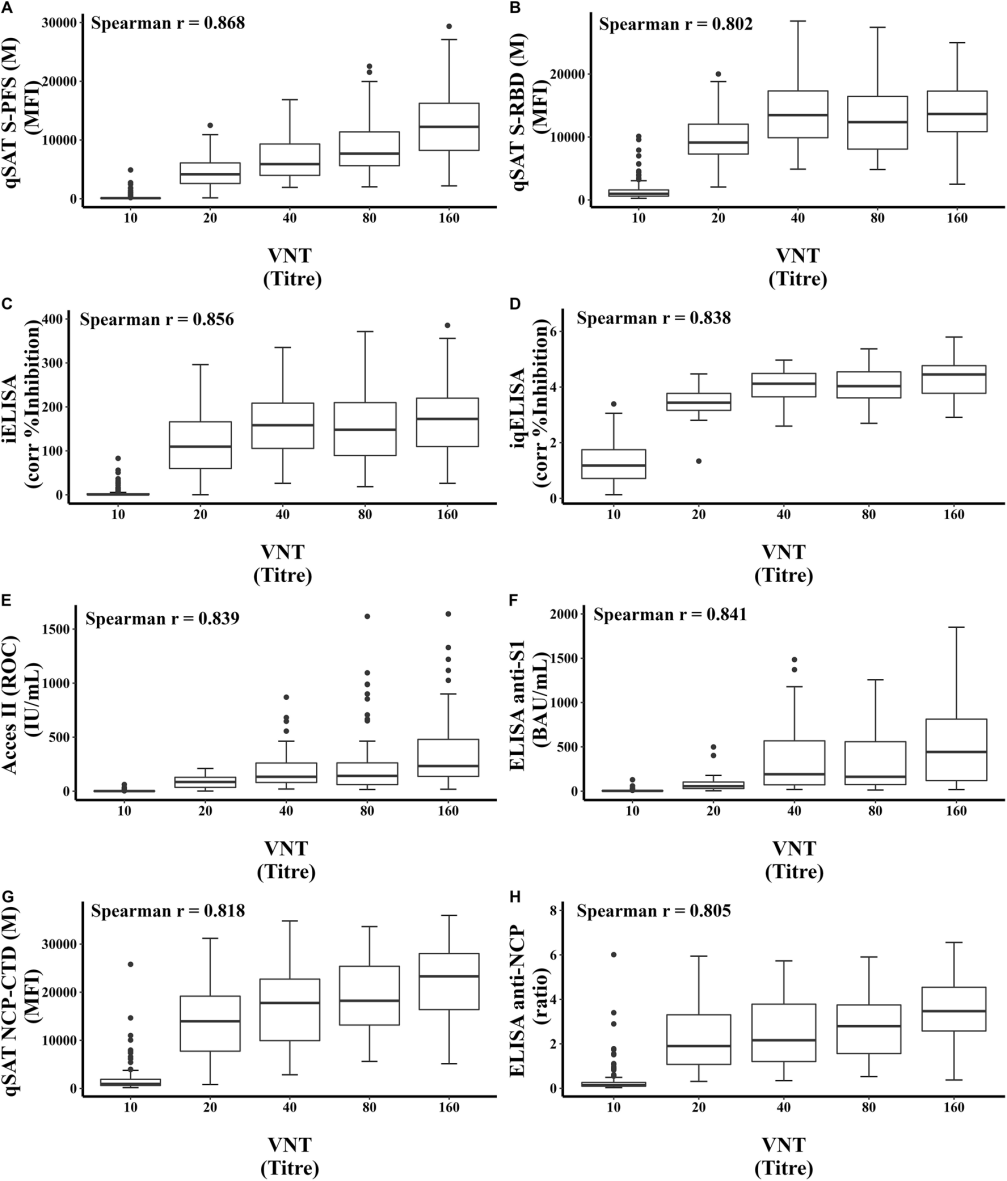
Correlation of cell-free serological tests with the SARS-CoV-2 viral neutralization test. Comparison of all cell-free serological assay with VTN were assessed through a Spearman correlation test. *P* value for each comparison was < 0.001.

The extent to which the different cell-free serological tests could be equivalent to VNT was also assessed by a Spearman correlation (Fig. 3). In agreement with the analyses above, all serological tests showed strong positive correlations with VNT (Spearman r ranging from 0.802 to 0.868), indicating that they perform adequately in predicting the level of neutralizing antibodies. The Luminex assay using the S-PFS antigen showed the best correlation with VNT, even better than with the RBD antigen. The iELISA/iqELISA and Beckman’s Access SARS-CoV-2 IgG II assay showed excellent correlation with VNT, as expected, since they are designed to detect anti-RBD antibodies and that most of neutralizing antibodies target RBD.

### Evaluation of the qSAT assay on vaccinated samples

To evaluate the interest of the qSAT assay in a vaccinated or partially-vaccinated population, we used a Healthcare Workers (HCW) panel (cf. Methods) containing 813 samples collected in France. This panel from individuals vaccinated with one, two or three doses, was characterized by the “ELISA + VNT” strategy and a the qSAT assay using the Spike (S-PFS and S-RBD) and non-Spike antigens (NCP-CTD) (Table 1). The use of the Spike antigens revealed a high prevalence (> 96%) of either vaccination or infection within this population, aligning with observations made through the “ELISA + VNT” strategy and as expected for a vaccinated cohort. Non-Spike antigens unveiled the existence of antibodies generated solely in response to infection, indicating prevalence rates of 37.4%, 12.4%, and 10.0% for individuals vaccinated with one, two, or three doses, respectively.

**Table 1:** Evaluation of Healthcare workers panel by the “VNT + ELISA” strategy and the qSAT assay.

Test	Status	1 Vaccine dose N (%)	2 Vaccine doses N (%)	3 Vaccine doses N (%)
ELISA anti-S1	Negative	1 (0.5%)	0 (0%)	0 (0%)
	Equivocal	5 (2.5%)	3 (0.5%)	0 (0%)
	Positive	192 (97.0%)	592 (99.5%)	20 (100.0%)
VNT	Negative	17 (8.6%)	8 (1.3%)	0 (0%)
	Positive	181 (91.4%)	587 (98.7%)	20 (100.0%)
qSAT S-PFS (M)	Negative	6 (3.0%)	3 (0.5%)	0 (0%)
	Positive	192 (97.0%)	592 (99.5%)	20 (100.0%)
qSAT S-RBD (M)	Negative	0 (0%)	1 (0.2%)	0 (0%)
	Positive	198 (100.0%)	594 (99.8%)	20 (100.0%)
qSAT NCP-CTD (M)	Negative	124 (62.6%)	521 (87.6%)	18 (90.0%)
	Positive	74 (37.4%)	74 (12.4%)	2 (10.0%)

## Discussion

Here, the performance of two inhibition/competition ELISAs (iELISA/iqELISA), based on commercial ELISA kits, a chemiluminescence immunoassay, both designed to detect anti-RBD antibodies, as well as a qSAT assay using SARS-CoV-2 Spike full length and RBD domain, and NCP proteins as antigens were evaluated. The development of inhibition/competition assays capable of detecting anti-RBD antibodies has improved the sensitivity of the original anti-Spike S1 ELISAs regardless of the cut-off used, validating the value of using such assays as first-line screening. Concerning the qSAT assay, the performances obtained with S-PFS antigen proved to be better than with RBD and NCP-CTD antigens. Indeed, the S-PFS protein has several mutations that increase the RBD accessibility to nAbs (30% of the molecules have two RBD in an ‘down’ state and 70% have one RBD in an ‘up’ state)^33^. This pre-fusion conformation, which represents the receptor-accessible state that allows binding to ACE2^15,18^ and features quaternary epitopes naturally recognized by the immune system, probably leads to a better specificity of the test. Furthermore, the simultaneous use of RBD and S-PFS antigens gives a chance for all possible epitopes to be recognized, such as quaternary and S-NTD epitopes only present in S-PFS, or fully available RBD/RBM epitopes in the RBD antigen that would be hidden in the trimeric Spike.

The iELISA/iqELISA, Beckman's Access SARS-CoV-2 IgG II assay or our Luminex assay had sensitivities and specificities above 95%, were equally effective in detecting anti-RDB antibodies, and were highly correlated with each other and with VNT. Altogether, our results tend to show that all of these assays could potentially serve as alternatives to the “ELISA + VNT” strategy and therefore be used to determine the seroprevalence of SARS-CoV-2, at least in cohort with high expected prevalence, without the use of the laborious, highly technical seroneutralization assay requiring a level two or three biosafety laboratories (BSL2 or BSL3).

The RBD domain of SARS-CoV-2 Spike allows the virus to interact with its ACE2 receptor and thereby control the entry of the virus into the cell^15^. Furthermore, the RBD is the major immunodominant site of the Spike protein and anti-RBD antibodies are most involved in viral neutralization as previously shown^20,21^. Therefore, some cell-free assays targeting the ability of anti-RBD antibodies to prevent/neutralize the interaction of the Spike protein with its ACE2 receptor give results equivalent to VNT^34^. Here, using cell-free assays capable of specifically detecting anti-RBD antibodies, we have shown that direct detection of anti-RBD antibodies also correlates with VNT results. This reinforces the idea that anti-RBD antibodies are antibodies preferentially involved in viral neutralization and explains why direct detection of anti-RBD antibodies could be used instead of VNT, at least for seroprevalence studies.

Using NCP antigen could allow the detection of infection and distinguish from vaccination. However, anti-NCP antibodies are expected to persist for a shorter time than anti-Spike antibodies^35^. Therefore, some non-NCP positive but Spike-positive samples might belong to individuals who were previously infected and have subsequently lost their antibodies, as described before, or could genuinely be individuals who were never infected. Moreover, this approach is entirely suitable for regions where exclusively Spike-expressing vaccines, such as Pfizer's or Moderna’s, are widespread, notably in regions like Europe and North America. However, its efficacy is compromised in countries that have also implemented SARS-CoV-2 inactivated vaccines, as seen in Asia, Africa, and South America.

In addition, the iELISA/iqELISA and Luminex methods are ideally suited to meet the massive needs to analyze the serological status of samples from very large cohorts. Beckman's Access SARS-CoV-2 IgG II assay has a lower throughput (approximately 1000 samples/day versus 200 samples/day) but could also be used for seroprevalence studies. The iELISA/iqELISA methods require only common equipment and instrumentation, making the methods usable in any laboratory in the world capable of performing an ELISA, unlike the Beckman's Access SARS-CoV-2 IgG II, Luminex and VNT tests that require specific equipment and/or facilities. Several commercial ELISAs for surrogate virus neutralization have also been developed^34^. These tests, based on antibody-mediated blockage of the ACE2-Spike protein–protein interaction, correlate well with virus neutralization tests and do not require the use of live viruses or BSL2 or BSL3 facilities. Nevertheless, they remain much more expensive than an anti-Spike ELISA and their availability in middle- and low-income countries could be a problem. The iELISA/iqELISA presented in this study also correlates well with VNT and could circumvent the problems associated with commercial surrogate virus neutralization ELISAs, as it is highly versatile and can be implemented on any commercial or in-house ELISAs targeting the SARS-CoV-2 Spike protein.

The “ELISA + VNT” strategy that we previously used was based on a first anti-Spike S1 ELISA screening^31,32^. To ensure optimal sensitivity of the anti-Spike S1 ELISA screen, we had decided to lower the manufacturer’s recommended ELISA cut-off ratio from 0.8 to 0.7 (from 35.2 to 18 BAU/mL) and to perform VNT on all samples with ratios > 0.7. This strategy was validated by the present results since the ROC analysis showed that the optimal sensitivity and specificity were reached using cut-off ratio of 0.75 or 18.4 BAU/mL. In addition, our results showed that anti-NCP ELISA displayed similar sensitivity to anti-Spike S1 ELISA (even using cut-off from ROC analysis), thus suggesting that anti-NCP ELISA could have been used as a first line screening.

As SARS-CoV-2 mutations have continued to emerge and spread, several studies have demonstrated reduced neutralization of the variants by post-vaccination and convalescent sera, as well as therapeutic monoclonal antibodies^36^. In this scenario of constant evolution, the question of the real validity or efficacy of using SARS-CoV-2 Wuhan-derived antigens containing assays to detect antibodies elicited against newer variants like those from Omicron lineage is still to be answered. In particular, whether the assays able to detect infection of unvaccinated people with Omicron variants. Here, we used samples from vaccinated individuals gathered between January 2021 and January 2022 in metropolitan France. This timeframe corresponds to a period during which the Alpha, Beta, Delta, and Omicron BA.1 variants were in circulation. The outcomes of the qSAT assay, using SARS-CoV-2 Wuhan-derived antigens, correlated with the VNT results. This implies that the assay is appropriate for seroprevalence studies, possibly even for recent variants like Omicron-derived ones. Indeed, since new variants acquire mutations primarily in the RBD domain, the use of the entire spike protein from Wuhan strain as an antigen (S-PFS) is expected to remain a less affected target than the RBD domain after infection with one of the latest mutants. Therefore, choosing S-PFS should at least ensure the accurate determination of the sample's positive or negative status. Nevertheless, strictly speaking, the qSAT assay does not provide information regarding the existence and strength of neutralizing antibodies against different variants that were circulating during the observed period. Indeed, in specific scenarios where accurate assessment of neutralizing antibody presence and quantity for each variant is necessary, like evaluating vaccine or prophylactic therapy immunogenicity or determining the immune status of vulnerable populations susceptible to severe COVID-19, the seroneutralization method remains the gold standard, notwithstanding the limitations mentioned in the introduction. Thus, the possibility of a rapid serological test that could detect emerging variants as they appear or predict the decrease in neutralization activity is very appealing, and could, even in the case of the scenarios mentioned above, provide complementary or confirmatory information to the seroneutralization method (see below). This possibility could be considered by an iELISA/iqELISA or by a qSAT assay by adding the RBD antigens corresponding to the variants of interest. A multiplex qSAT assay that includes all variant RBD’s could also be used for seroprevalence studies enabling the possible detection of antibodies produced against variants circulating in a particular area. While it is technically feasible with the qSAT assay, assessing the performance of a test incorporating the RBD corresponding to various SARS-CoV-2 variants could be a highly complex task. Indeed, this evaluation requires samples with comprehensive data on vaccination and infection specific to each variant to determine the specificity for each RBD antigen. However, the access to such samples for every newly emerging variant remains challenging. To date, no commercial test allows the distinction of the immune response produced against the SARS-CoV-2 variants.

Finally, the cell-free serological assays presented here have been shown to be effective for conducting seroprevalence studies, not only for detecting previous infection or vaccination, but also for providing a throughput that is suitable for large cohorts, at a controlled cost, and that can remain feasible in a standard laboratory. However, this type of test, with the advantage of specifically detecting anti-RBD antibodies, could also be developed for other applications in combination with classical serological methods that measure all antibodies recognizing the spike protein and/or VNT. This could, for example, provide important and quantitative information on the efficacy of antibody neutralization (especially in people with low antibody levels), the type of antibodies involved (IgG, IgM, IgA), the role of mutations present in the RBD as new variants emerge. The development of cell-free assays that specifically detect antibodies that are directed away from the RBD but are expected to neutralize the virus, e.g. against the N-terminal domain of Spike, could also be used in the future to more systematically investigate the role of these antibodies in neutralization of the new variants in comparison to anti-RBD antibodies.

In conclusion, based on the current state of knowledge derived from our study, we recommend the cell-free tests presented here, especially the qSAT assay, for conducting seroprevalence studies.

## Methods

### Specimens

Four different groups of samples were used in our study:

1. Epidemic panel: plasma samples (N = 486) collected in April 2020 in Metropolitan France from volunteer blood donors that include 230 samples from donors who previously had a PCR-proven SARS-CoV-2 infection and declared the disappearance of symptoms at least 14 days prior to donation, and 256 samples from donors who declared no symptoms or previous SARS-CoV-2 infection. These samples were part of a study for the establishment of a COVID-19 Convalescent Plasma (CCP) collection that was approved by the ethics review committee of the French National Agency for Medicines and Health Products Safety (ANSM) (N° IDRCB = 2020-A00728-31).
2. Pre-epidemic panel: plasma samples (N = 180) from healthy adult French blood donors collected in 2018 (COVID-19 pre-pandemic panel). All donors were informed that data and samples associated with their donation could be used for research purpose.
3. A pool of 4 sera samples with a high anti-SARS-CoV-2 antibody content from Bolivian volunteer blood donors from the Tarija blood bank. The pool was obtained by mixing 100µL of each sample. Sample collection and protocol were approved by the ethics committee of the Dr. Mario Ortíz Suárez hospital, Santa Cruz de la Sierra, Bolivia (N° FWA0002686).
4. Healthcare Workers (HCW) panel: sera samples (N = 813) collected from January 2021 to January 2022 in Metropolitan France from Healthcare Workers after vaccination with one, two or three doses of Comirnaty (Pfizer & BioNTech) vaccine. Sample collection was approved by the “Sud-Ouest et Outre-Mer II” ethics review committee (N° IDRCB = 2020-A01653-36).

Informed consent was obtained from all subjects and only donations from donors who consented to research use were included in the study. All methods were performed in accordance with the relevant guidelines and regulations.

### Virus neutralization test (VNT)

Virus Neutralization Tests (VNT) were performed as previously described^31,32^. Briefly, the test consists of mixing 110 µL of a serially diluted (from 1/20 to 1/160) patient sample in DMEM with 1% of penicillin/streptomycin, Non-Essential Amino Acids and Glutamine, with 110 µL of a fixed quantity of SARS-CoV-2 strain BavPat1 (courtesy of Prof. Drosten, Berlin) corresponding to 0.5 TCID_50_/µL of plasma dilution. This mixture is then incubated for 1 h at 37 °C. One hundred µL of the mixture was then transferred onto a confluent Vero E6 TMPRSS2 + cells monolayer and incubated at 37 °C under 5% of CO_2_. On day 5 post infection, dilutions showing a cytopathic effect (CPE) were considered negative (no neutralization) and those without CPE were considered positive (complete neutralization of the SARS-CoV2 inoculum). The neutralization titer referred to as the highest serum dilution with a positive result. Specimens with a VNT titer ≥ 20 were considered as positive.

### ELISA assays

The samples were screened using three commercial kits (Euroimmun) according to manufacturer's recommendations. Two generations of tests were assessed for the detection of IgG against the SARS-CoV-2 Spike S1 domain: one qualitative, the Anti SARS-CoV-2 ELISA IgG and one quantitative, the Anti SARS-CoV-2 Quantivac ELISA IgG. The third test used, a qualitative ELISA for the detection of IgG against the N protein, was the Anti-SARS-CoV-2 NCP ELISA IgG.

Regarding qualitative assays, specimens with ratio < 0.8, 0.8 ≤ ratio < 1.1 and ratio > 1.1 were considered negative, equivocal and positive, respectively. For the anti-S1 quantitative assay, boundaries were: BAU/mL < 25.6, 25.6 ≤ BAU/mL < 35.2, BAU/mL ≥ 35.2, respectively.

### Inhibition/competition ELISAs

To assess the presence of anti-RBD IgG antibodies in human plasma samples, we modified the commercial anti-SARS-CoV-2 Spike S1 ELISAs, one qualitative (ELISA anti-S1) and one quantitative (ELISA anti-S1 (Q)). The modified assays are hereafter referred to as iELISA and iqELISA, respectively. The presence of anti-RBD antibodies was assessed indirectly by inhibition of the anti-Spike S1 antibody signal, through the competition binding of anti-RBD antibodies to recombinant RBD protein.

Before performing the ELISA, a pre-incubation step at 37°C for 1 h of the sample with 2µg/mL recombinant RBD (Thermo Fisher Scientific) diluted in the kit sample buffer was added. Each sample was analyzed in parallel with and without RBD in the pre-incubation step. Absorbance was measured with a SUNRISE spectrophotometer (Tecan) at 450 nm with a reference wavelength at 620 nm. Results were determined as indicated by the manufacturer by calculating a ratio of sample OD to calibrator OD for the anti-SARS-CoV-2 Spike S1 ELISA IgG and the BAU/mL value for the anti-SARS-CoV-2 Spike S1 QuantiVac ELISA IgG. To account for samples with very high levels of anti-S1 antibodies showing low *%Inhibition* due to insufficient amount of RBD used, while minimizing the amount of RBD to be used, a correction for *%Inhibition* was introduced by multiplying it by the value of the direct anti-S1 signal (ratio or BAU/mL) and referred as *corr %Inhibition*. The corrected percentage of inhibition was calculated by using the following formula for the anti-SARS-CoV-2 Spike S1 ELISA IgG:

1 $$\begin{aligned} iELISA\;corr\% \;Inhibition & = Sample\;ratio\;without\;RBD*\left( {100 - \left( {100*\left( {Sample\;ratio} \right.} \right.} \right. \\ & \quad \left. {with\;RBD} \right)/\left. {\left. {\left( {Sample\;ratio\;without\;RBD} \right)} \right)} \right) \\ \end{aligned}$$

and for the anti-SARS-CoV-2 Spike S1 QuantiVac ELISA IgG:

2 $$\begin{aligned} iqELISA\;corr\% \;Inhibition & = BAU/mL\;value\;without\;RBD*\left( {100 - \left( {100*\left( {BAU/mL} \right.} \right.} \right. \\ & \quad \left. {value\;with\;RBD} \right)/\left. {\left. {\left( {BAU/mL\;value\;without\;RBD} \right)} \right)} \right) \\ \end{aligned}$$

### Access SARS-CoV-2 IgG II assay

The commercial Access SARS-CoV-2 IgG II (Beckman Coulter) chemiluminescent immunoassay was used as a comparative test intended for the semi-quantitative determination of IgG antibodies to SARS-CoV-2 receptor binding domain (RBD). The amount of anti-SARS-CoV-2 RBD IgG antibodies in the sample is determined from a stored multipoint calibration curve. All tests were conducted according to the manufacturer's recommendations. A cut-off of 30 IU/mL was used for positivity as recommended by the manufacturer.

### Luminex qSAT assay

Three commercial proteins produced in HEK293 cells expression system, and two N proteins available at the European Viral Archive goes global (EVAg) repository (www.european-virus-archive.com) were selected for the qSAT assay: (1) the trimeric extracellular domain (ECD) (aa 16-1213) of the SARS-CoV-2 spike glycoprotein (S) (Sino Biological); (2) the trimeric extracellular domain (ECD) (aa 16-1213) of S protein in its prefusion form (bearing the mutations R683A, R685A, F817P, A892P, A899P, A942P, K986P and V987P, Sino Biological); (3) the Receptor-Binding Domain (RBD) (aa 319-541) of S protein (Thermo Fisher Scientific); (4) the NTD domain of the SARS-CoV-2 N protein (EVAg, ref: 100P-03956); (5) the CTD domain of the SARS-CoV-2 N protein (EVAg, ref: 100P-03957).

Each antigen (60 pmol/10^6^ beads) was coupled to a distinct region of MAGPLEX^®^ magnetic microspheres (Luminex Corporation) using the xMAP^®^ Antibody Coupling Kit (Luminex Corporation) following manufacturer’s recommendations. The coupled beads were resuspended and counted on a Countess™ II Automated Cell Counter (Thermo) to a final concentration of 2 × 10^6^ bead/ml. Samples diluted in Wash Solution (Thermo) at 1/400 were incubated with 1000 coupled beads per well for 1 h at room temperature in a plate shaker protected from light. After two washes, the beads were incubated with R-Phycoerythrin AffiniPure F(ab')_2_ Fragment Goat Anti-Human IgG (H + L) (Jackson ImmunoResearch) for 1 h at room temperature in a plate shaker protected from light. After washing, antigen–antibody reactions were read on a MAGPIX^®^ system using the xPONENT^®^ software (Luminex Corporation) and the results were expressed as median fluorescence intensity (MFI). To determine IgG binding antibody units (BAU/mL), the pool of sera samples was threefold serially diluted and each dilution was quantified by ELISA. Each quantified dilution was used a standard in all qSAT experiments to convert the MFI results in BAU/mL.

### “ELISA + VNT” strategy

All specimens were primarily analyzed with the commercial Anti SARS-CoV-2 Quantivac ELISA IgG and Anti-SARS-CoV-2 NCP ELISA IgG from Euroimmun. The non-negative samples (Positive and equivocal with cut-off ratio > 0.7) were then tested by the virus neutralization test (VNT). The status of each sample was defined as positive when the VNT result was positive and/or both anti-S1 and -NCP ELISAs were positive.

### Statistical analysis

Statistical analysis was performed using RStudio version R-4.0.3 through the ROCR and cutpointr packages^37,38^. Inhibition ELISA cutoff values, accuracy, sensitivity and specificity were calculated using receiver operating characteristics (ROC) curve analysis. Correlations between all the tests were analyzed using a Pearson correlation test and linear regression model.

### Supplementary Information

Supplementary Information.

## Data Availability

The data presented in this study are available within the article.
