# Supplementary material for: Exploring cell-free assays for COVID-19 serosurvey
Source: Sci Rep. 2024 Mar 13;14:6096. doi: 10.1038/s41598-024-55852-6 (PMC10938000; doi:10.1038/s41598-024-55852-6)
Supplement: Supplementary file 1 — Supplementary Information. [file 41598_2024_55852_MOESM1_ESM.docx]

**Exploring cell-free assays for COVID-19 serosurvey**

**Authors**

Lucia Inchauste ^1^, Elif Nurtop ^1^, Nadège Brisbarre ^1,2^, Laetitia Ninove ^1^, Pierre Gallian ^1,3^, Xavier de Lamballerie ^1^ and Stéphane Priet ^1^

**Affiliations**

^1^ Unité des Virus Émergents (UVE: Aix-Marseille Univ, Università di Corsica, IRD 190, Inserm 1207, IRBA), Marseille, France.

^2^ Établissement Français du Sang Provence Alpes Côte d'Azur et Corse, Marseille, France.

^3^ Établissement Français du Sang, La Plaine Saint-Denis, France.

Address correspondence to Stéphane Priet, stephane.priet@univ-amu.fr

**Supplementary information**

**Supplementary Table 1:** Tests performances: Sensitivity (Se), Specificity (Sp), Positive and Negative samples and count of True Positives (TP), False Negatives (FN), False Positives (FP) and True Negatives samples.

**Supplementary Figure S1:** Empirical test performance indicators.

**Supplementary Figure S2:** Correlation analysis between singleplex and multiplex qSAT assays for S-RBD (A), S-PFS (B) and NCP-CTD (C) antigens.

**Supplementary Figure S3:** Side-by-side comparisons between multiplex qSAT assays for S-RBD, S-PFS and NCP-CTD antigens.

**Supplementary Figure S4:** Correlation between BAU/mL transformation for the qSAT S-PFS antigen and the quantitative ELISA anti-S1.

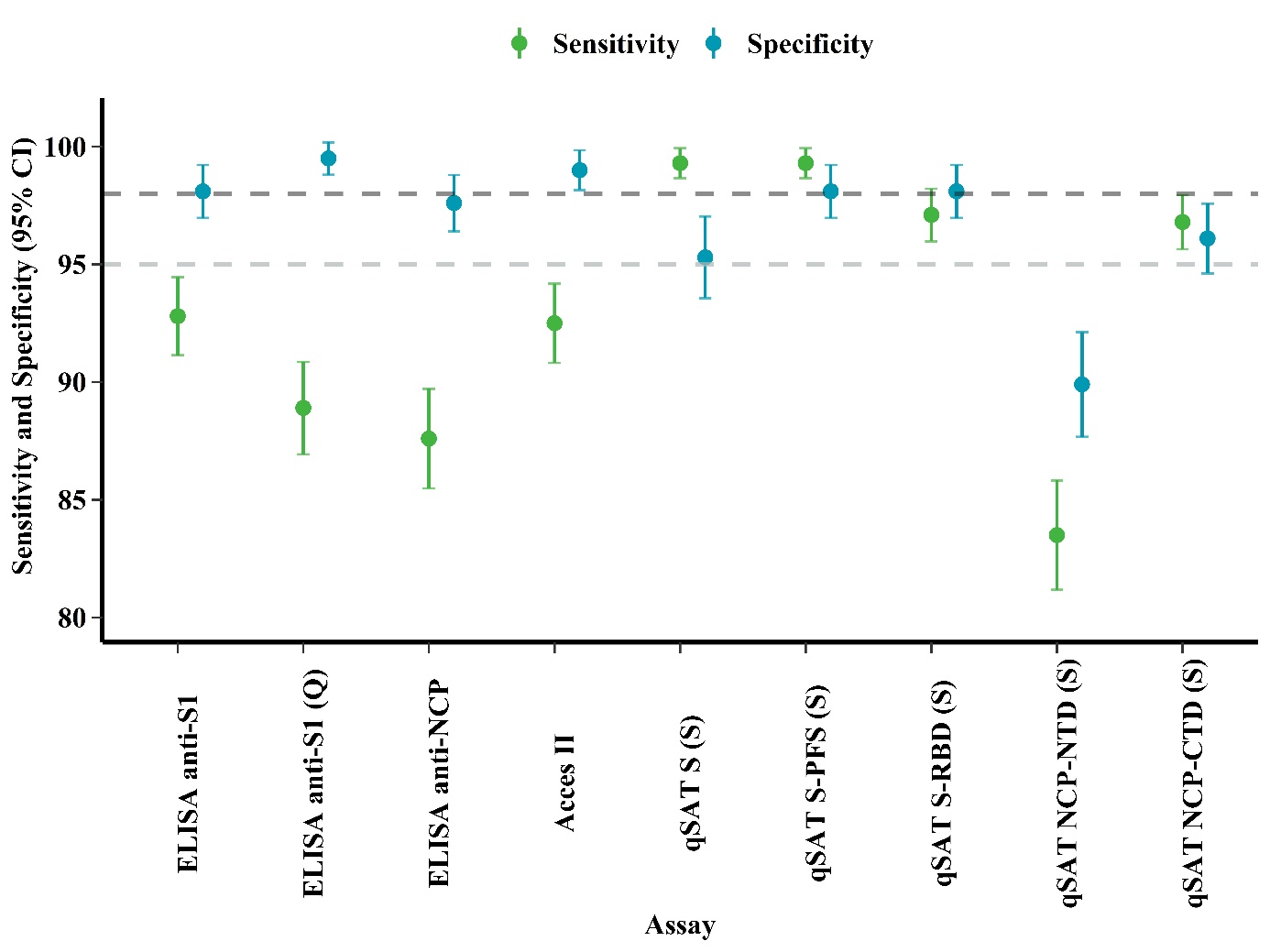


**Supplementary Figure S1: Empirical test performance indicators.**

Sensitivity (green dots), specificity (lightblue dots) and respective 95% confidence intervals (95%CI, error bars) were calculated either with the manufacturer cut-off value for the ELISA’s and the CLIA assays or by Receiver Operating Characteristic curve (ROC) analysis for the qSAT assay.


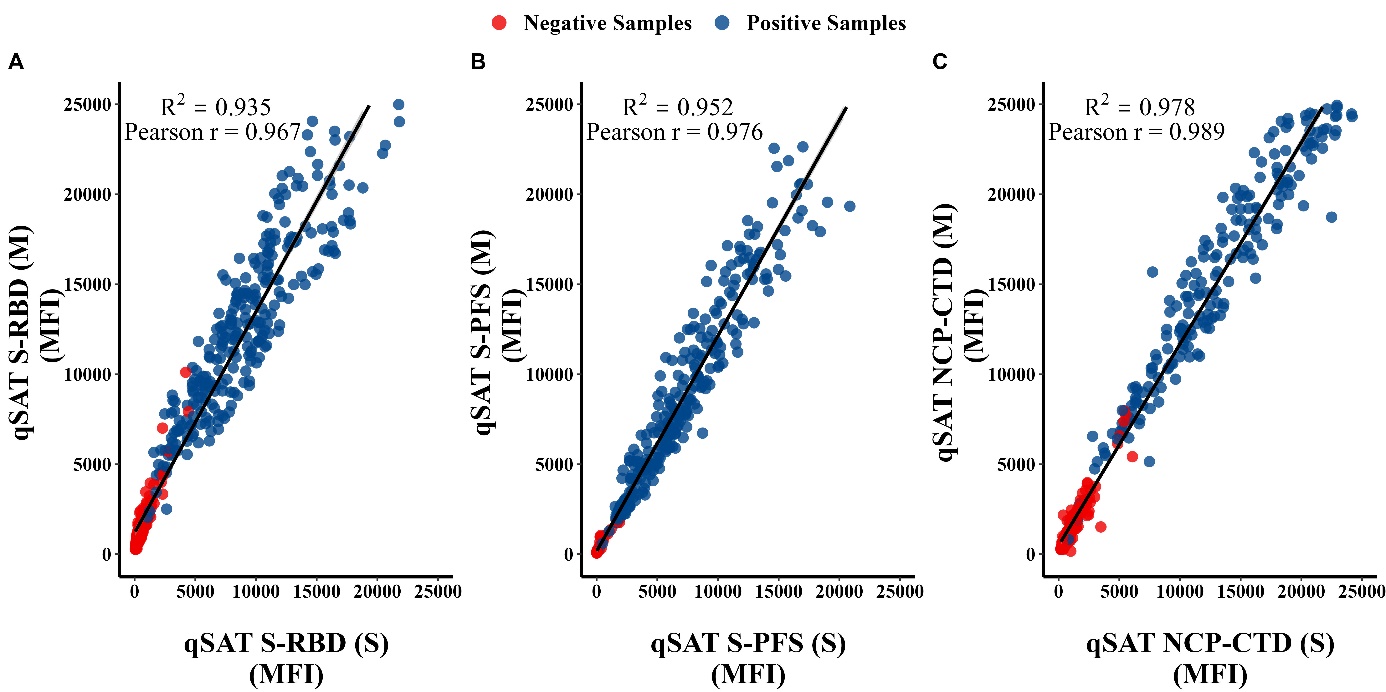


**Supplementary Figure S2: Correlation analysis between singleplex and multiplex qSAT assays for S-RBD (A), S-PFS (B) and NCP-CTD (C) antigens.**

Samples were considered positive (blue dots) with either a positive VNT result or a positive result for both the anti-Spike S1 and -NCP IgG ELISA, others were considered negative (red dots). Correlations were assessed through a Pearson analysis. P-value for each comparison was <0.001.


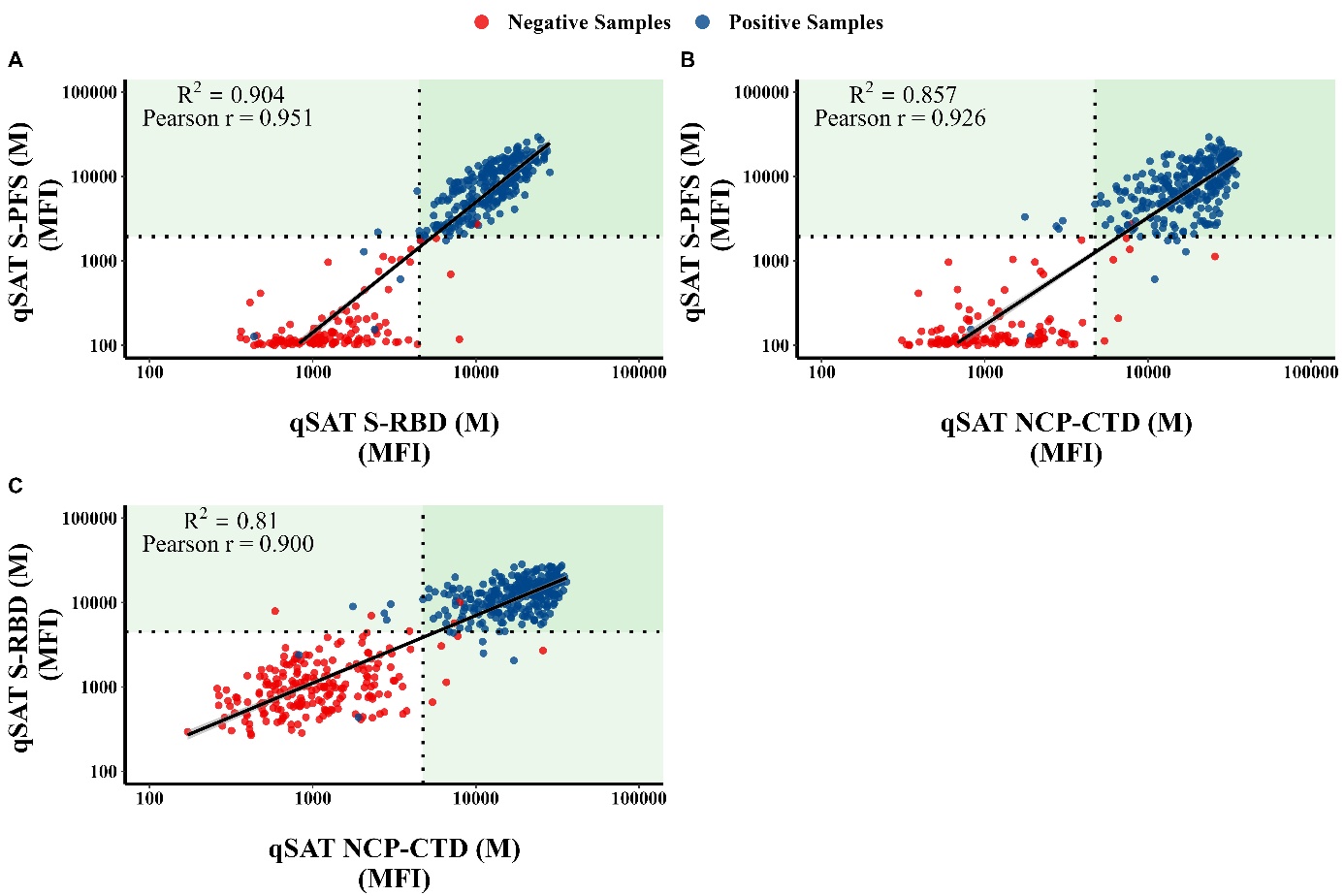


**Supplementary Figure S3: Side-by-side comparisons between multiplex qSAT assays for S-RBD, S-PFS and NCP-CTD antigens.**

Samples were considered positive (blue dots) with either a positive VNT result or a positive result for both the anti-Spike S1 and -NCP IgG ELISA, others were considered negative (red dots). Side-by-side comparisons were assessed through a Pearson correlation test. P-value for each comparison was <0.001.


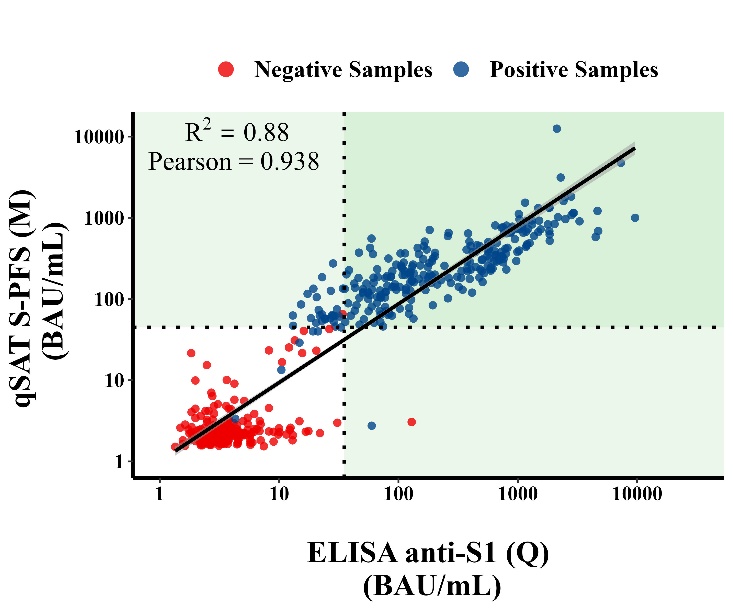


**Supplementary Figure S4: Correlation between BAU/mL transformation for the qSAT S-PFS antigen and the quantitative ELISA anti-S1.**

Samples were considered positive (blue dots) with either a positive VNT result and/ or a positive result for both the anti-Spike S1 and -NCP IgG ELISA, others were considered negative (red dots). Comparison was assessed through a Pearson correlation test. P-value was <0.001.
